# Time-Resolved Spectroscopic Study of *N*,*N*-Di(4-bromo)nitrenium Ions in Selected Solutions

**DOI:** 10.3390/molecules23123182

**Published:** 2018-12-03

**Authors:** Lili Du, Xin Lan, Zhiping Yan, Ruixue Zhu, David Lee Phillips

**Affiliations:** 1 Department of Chemistry, The University of Hong Kong, Hong Kong S.A.R., China; justailleen@gmail.com (L.D.); xinlan@connect.hku.hk (X.L.); mcayzp@gmail.com (Z.Y.); zhurx@shanghaitech.edu.cn (R.Z.); 2 Institute of Life Sciences, Jiangsu University, Zhenjiang 212013, China; 3 School of Physical Science and Technology, Shanghai Tech University, Shanghai 201210, China

**Keywords:** nitrenium ion, resonance Raman, transient absorption, reactive intermediate

## Abstract

Nitrenium ions are important reactive intermediates in chemistry and biology. In this work, femtosecond and nanosecond transient absorption (fs-TA and ns-TA) along with nanosecond time-resolved resonance Raman (ns-TR^3^) experiments were employed to examine the photochemical pathways of *N*-(4,4′-dibromodiphenylamino)-2,4,6-trimethylpyridinium BF_4_^−^ (salt (DN) from just absorption of a photon of light to the production of the important *N*,*N*-di(4-bromophenyl)nitrenium ion **2**. In acetonitrile (MeCN), the formation of halogenated diarylnitrenium ion **2** was observed within 4 ps, showing the vibrational spectra with strong intensity. The nucleophilic adduct reaction of ion **2** with H_2_O was also examined in aqueous solutions. The direct detection of the unique ortho adduct intermediate **3** shows that there is an efficient and exclusive reaction pathway for **2** with H_2_O. The results shown in this paper give new characterization of **2**, which can be used to design time-resolved spectroscopy investigations of covalent addition reactions of nitrenium ions with other molecules in future studies.

## 1. Introduction

Nitrenium ions are important intermediates which structurally have a formal positive charge on the nitrogen. Many research works have shown that aryl nitrenium ions have mutagenic properties in chemical carcinogenesis of aromatic amines [1,2,3,4,5,6,7,8,9].

**Scheme 1:**
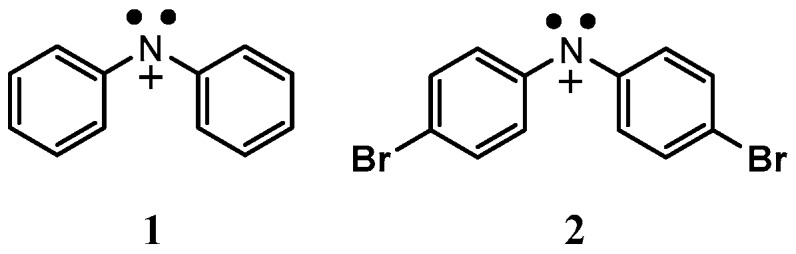
The structures of nitrenium ions.

**Scheme 2:**
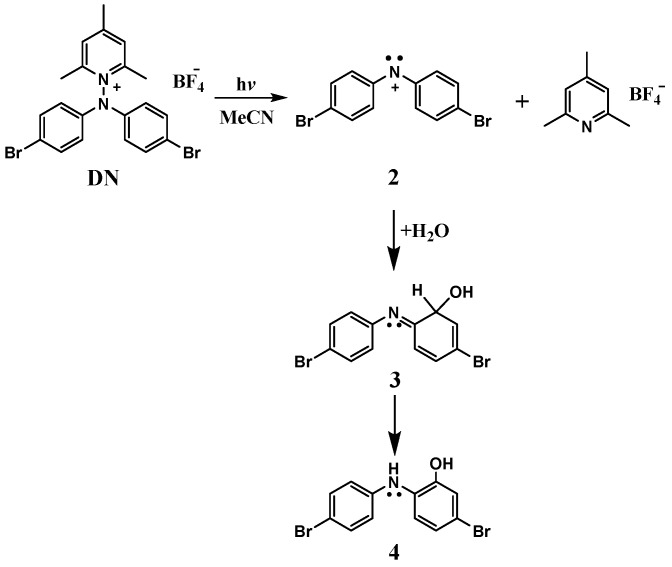
Reaction mechanism for photoexcitation of *N*-(4,4’-dibromodiphenylamino)-2,4,6-trimethylpyridinium BF_4_^−^ salt (DN) proposed by Falvey and coworkers [10].

Falvey and coworkers [10,11,12,13] reported several works on the chemical and spectroscopic behavior of the *N*,*N*-diphenylnitrenium ion (**1**) formed after photoexcitation of the 1-(*N*,*N*-dipheylamino)-2,4,6-triphenylpyridinium ion (see Scheme 1), and this aryl nitrenium ion was proven to have a ground state singlet character with some overlap between an unfilled nitrogen based *p*-orbital with filled π-orbitals on the phenyl groups [12,14]. Furthermore, Thomas and Falvey’s research work showed that the *N*,*N*-di(4-bromophenyl)nitrenium ion (**2**) formed after photoexcitation of *N*-(4,4′-dibromodiphenylamino)-2,4,6-trimethylpyridinium BF_4_^−^ salt (DN) had greater stability with a longer lifetime than the unsubstituted nitrenium ion **1**, which was revealed by study of laser flash photolysis to have a lifetime of 124 µs in MeCN, and 6.57 µs in water [10]. Macernis et al., studied the effect of surrounding polar environments on the molecular behavior of the related nitrogen [15]. Even though the differing decay pathways of halogenated diarylnitrenium ion **2** in organic and aqueous solutions were predicted in some research as shown in Scheme 2, the reaction mechanisms of **2** remained unclear due to the lack of direct evidence regarding their structure and properties of the relevant intermediates [10].

In this paper, femtosecond transient absorption (fs-TA) was utilized to directly investigate the photo-induced formation of halogenated diarylnitrenium ion **2**. In addition, the structures and characteristics of **2** were characterized with nanosecond time-resolved resonance Raman (ns-TR^3^) spectroscopy experiments, and these results are probably the first time-resolved vibrational spectroscopic characterization of the halogenated diarylnitrenium ion **2**. Furthermore, nanosecond transient absorption (ns-TA) and ns-TR^3^ experiments were employed to examine the behavior of **2** in near neutral aqueous solution, and these results provide new insight into the structure and chemical reactivity of **2** in aqueous solutions.

## 2. Results and Discussion

### 2.1. In MeCN

**Figure 1:**
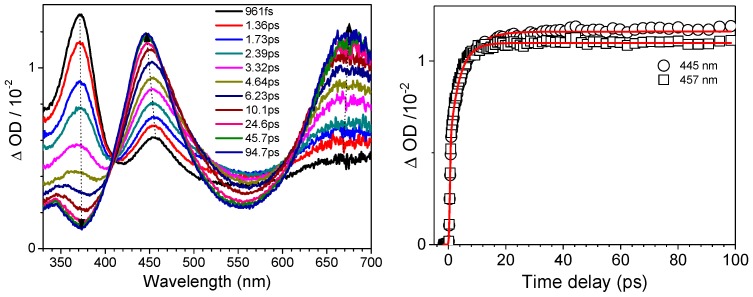
Displayed are femtosecond transient absorption (fs-TA) spectra of DN in acetonitrile (MeCN) acquired after 267 nm irradiation (**left**), and kinetics of the band at 445 nm and 457 nm (**right**).

Figure 1 displays the fs-TA spectra of DN in MeCN, and the early time spectra appearing at 370 nm and 457 nm may be attributed to the transient absorption of DN in its lowest excited singlet state. Later, the feature at 370 nm decreases in intensity significantly, with the 457 nm feature increasing in intensity and blue-shifting to 445 nm up to about 94.7 ps. Concurrently, the broad feature peaking at 675 nm appears and reaches its intensity maximum around 100 ps. The spectral variation from 1 ps to 100 ps can be attributed to indicate the generation of **2**, subsequent to photo-cleavage of the N-N bond, because it was also observed in nanosecond laser flash photolysis [10]. As displayed in the right of Figure 1, the decay kinetics of the absorption band at 445 nm and 457 nm can be modeled well with a one-exponential function with a 4.3 ps time constant. Thus, the DN excited singlet state decay and the generation of **2** can be determined to be 4.3 ps, with one species evolving into the other, as evidenced by the isobestic points seen in the fs-TA data. Comparison of these results to those for the growth time constant obtained for generation of the 2-fluorenyl nitrenium ion (167 ps) [16] indicates the faster photo-cleavage seen for DN results in much faster generation of **2**, which accompanied by its high yield, suggests this photochemical method for formation of **2** has good promise in investigating the reactions of **2** with nucleosides and other substrate molecules.

**Figure 2:**
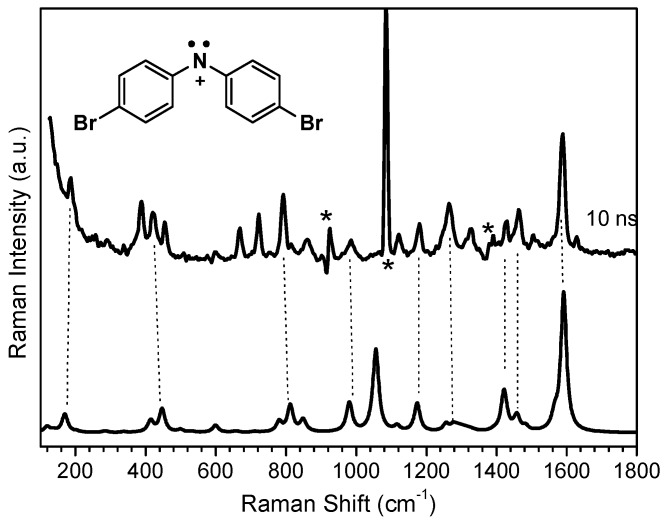
Comparison of the nanosecond time-resolved resonance Raman (ns-TR^3^) 10 ns spectrum obtained after excitation of DN in MeCN (pump 266 nm, probe 416 nm) to the density functional theory (DFT) calculated normal Raman spectrum of **2**. The symbol * means features affected by solvent subtraction artifacts.

So as to better understand the vibrational structural information and characteristics of **2**, ns-TR^3^ experiments were performed for DN in MeCN by using 266 nm for the photolysis and 416 nm for the wavelength of the probe. Figure S2 presents the ns-TR^3^ spectra obtained after photoexcitation of DN in MeCN from 10 ns up to 300 µs, and during this time region, mostly one intermediate was seen and its long lifetime is consistent with the literature value of the lifetime of **2**, found by Falvey and coworkers from their ns-TA study (124 µs) [10]. Density functional theory (DFT) computations were used to simulate the normal Raman spectrum of **2**, and comparison with the experimental TR^3^ spectrum is given in Figure 2. It can be seen that the predicted normal Raman spectrum for the nitrenium ion **2** agrees well with the 10 ns experimental TR^3^ spectrum based on comparison of the patterns of the vibrational frequencies. The small differences between the relative Raman intensities of the computed and experimental spectra could be due to the resonance enhancement for the experimental Raman spectra, whereas the computed one is a normal Raman spectrum without the resonance effect. There are several strong Raman features at 1589, 1463, 1264, 812, 447, and 169 cm^−1^ seen in the TR^3^ spectra, and those located at 1589, 1463, and 1264 cm^−1^ can mostly be attributed to C=C stretching vibrational modes of the phenyl rings. The 812 cm^−1^ Raman feature may be attributed to the H–C–C–H wagging vibrational modes. The features at 447 cm^−1^ and 169 cm^−1^ may be connected with the C–N–C bending mode and the C–Br stretching mode. The computations suggest the C–N stretching feature is located near 1371 cm^−1^ that is consistent with Falvey and colleagues literature report for the diphenylnitrenium ion **1** using TR-IR spectra [17]. Unfortunately, the strong Raman MeCN feature at 1372 cm^−1^ obscures the C–N stretching feature by solvent subtraction. It is interesting to note the differences between **2** and **1** by comparing their DFT computed normal Raman spectra in Figure S3. By correcting by the same scaling factor, the vibrational mode for the stretching of the diphenyl rings of **1** also shows the feature at 1589 cm^−1^ is the same as that for **2**. Intriguingly, the intensities of Raman features in the 200–1500 cm^−1^ region for the halogenated **2** intermediate have much greater relative intensity compared to the non-halogenated **1** intermediate that may be due to the halogen electronic effect on the in-plane C–H bending vibrations [18]. Thus, the **2** intermediate may be thought of being a better nitrenium ion for observing nucleophilic reactions with some nucleosides by utilizing ns-TR^3^ spectroscopy [9].

### 2.2. In Aqueous Solution

Falvey and coworkers found that the nucleophilic reaction of 2 with H_2_O was first order in terms of the [H_2_O], and measured a 1.12 × 10^4^ M^−1^s^−1^ second order rate constant [10]. In comparison to the very high chemical reactivity of the DNA bases [6,9,10], the longer lifetime and lower chemical reactivity of **2** toward H_2_O enables the ability to determine the lifetimes and chemical reactivity of **2** and similar nitrenium ions in aqueous environments. Thus, the spectroscopic investigation in aqueous media may be useful information when planning for further investigations of the reactions of **2** with other molecules in the future.

**Figure 3:**
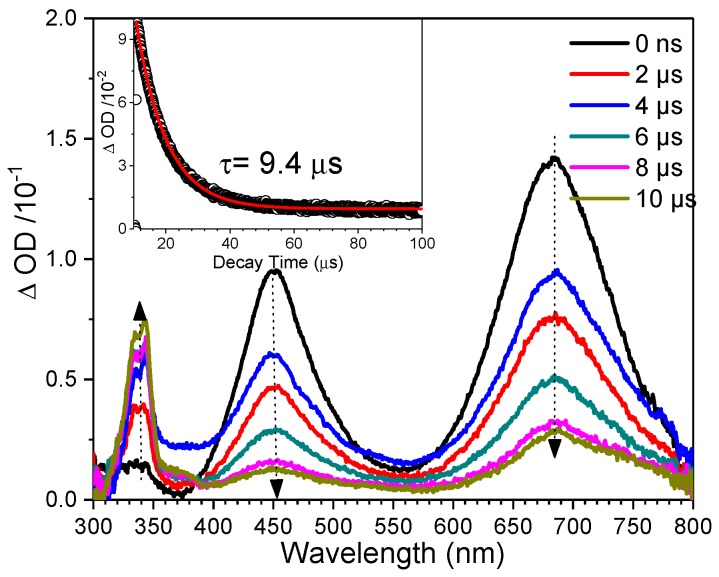
Shown are ns-TA spectra obtained in a 1:1 MeCN:H_2_O solution after 266 nm irradiation of DN. The kinetics at 450 nm appear in the insert.

The fs-TA spectra obtained after photoexcitation of DN in an aqueous solution are also shown in Figure S4, which were essentially the same as those seen in MeCN. The time constant for the formation of **2** in aqueous solution is also about 4 ps, which is very similar to that observed in MeCN. In addition, Figure 3 displays the ns-TA spectra obtained after photoexcitation of DN in aqueous solution. As the absorption features at 450 nm and 680 nm decay, the feature at 340 nm increases in intensity within about 9.4 µs. Because the para position of **2** is obstructed by bromine in comparison to **1**, one would expect the nucleophilic reaction to take place at the ortho positon of **2**, as seen in Scheme 2 [10]. Thus, the increasing intensity in the feature at 340 nm may be attributed to the formation of ortho adduct intermediate **3** after nucleophile addition or the final product **4**.

**Figure 4:**
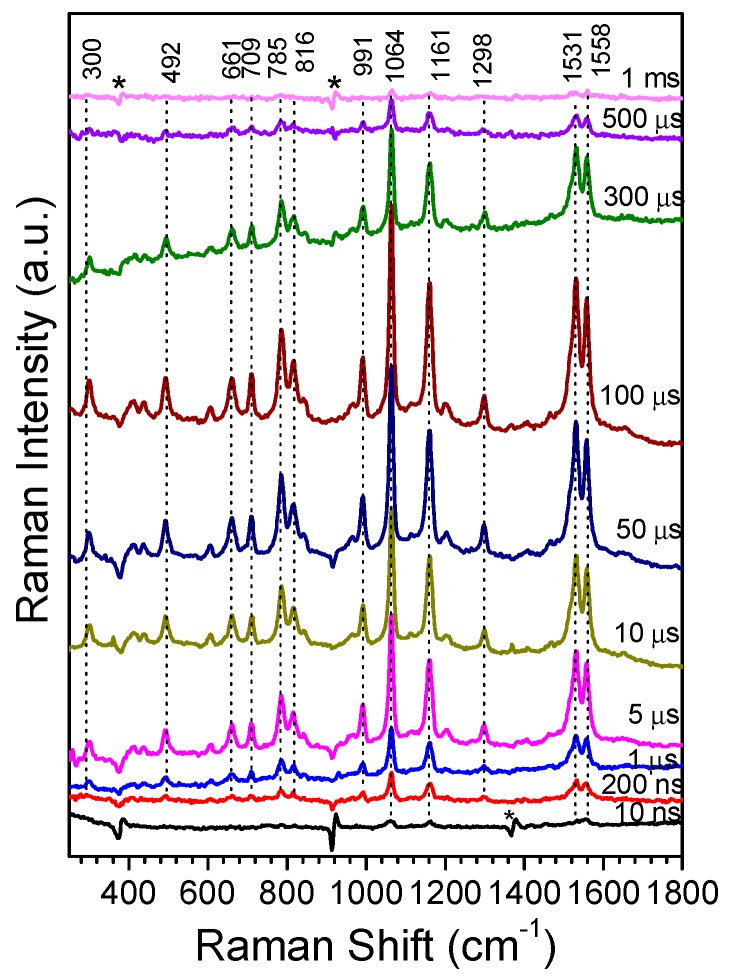
Shown are the ns-TR^3^ spectra using a 341.5 nm probe wavelength for intermediates formed after 266 nm photoexcitation of DN in a 1:1 MeCN:H_2_O. The star symbols mark places influenced by solvent subtraction artifacts.

**Figure 5:**
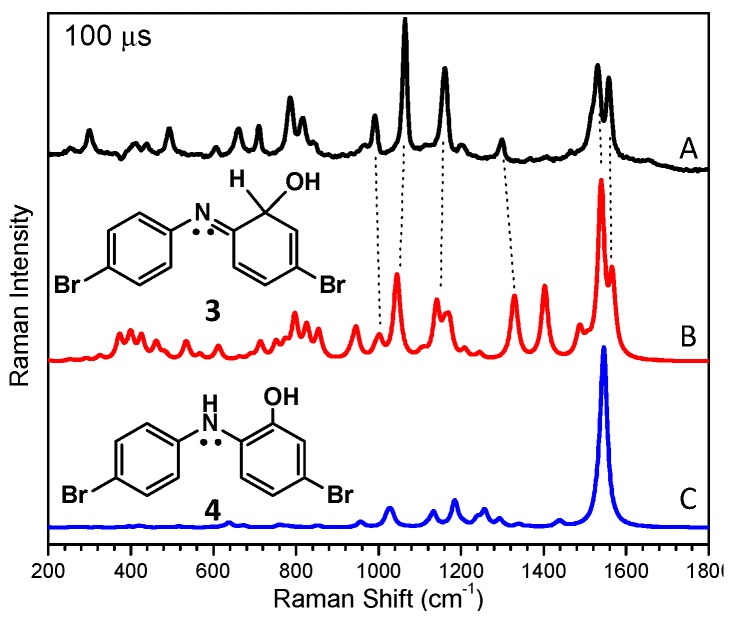
The ns-TR^3^ spectrum at 100 µs (A) obtained after 266 nm photoexcitation of DN in a 1:1 MeCN:H_2_O is compared with the DFT predicted normal Raman spectrum of intermediate **3** (B) and product **4** (C).

Ns-TR^3^ experiments were done to ascertain the vibrational properties of the involved intermediates in aqueous solution by using a probe laser wavelength of 341.5 nm and a 266 nm pump wavelength. According to the ns-TA spectra for intermediates observed after photoexcitation of DN in aqueous solution, the absorption features at 450 nm and 680 nm decay while the feature at 340 nm increases, which indicates that a new species is observed in aqueous solution that could be more easily detected by the ns-TR^3^ with a probe wavelength in the 300 nm to 370 nm region, and we thus selected 341.5 nm as a convenient probe wavelength to use from our experimental set-up. Figure 4 shows the ns-TR^3^ spectra obtained from 10 ns to 1 ms after 266 nm excitation of DN in a 1:1 MeCN: H_2_O solution. Inspection of Figure 4 shows a new species appears at 10 ns and reaches a maximum intensity near 100 µs, then decays over the next 900 µs. Figure 5 displays a comparison of the 100 µs ns-TR^3^ spectrum to the DFT predicted normal Raman spectrum of intermediate **3** and product **4** that clearly shows the computed vibrational frequencies and Raman intensities for intermediate **3** agree better with the experimental ns-TR^3^ spectrum vibrational frequency patterns. Thus, the longer lifetime intermediate seen in aqueous solution may be attributed to intermediate **3**. This assignment is in agreement with the mechanism proposed by Falvey and coworkers [10], and importantly gives a direct piece of evidence for the existence of nucleophilic adduct reaction intermediate **3** formed by reaction of **2** with H_2_O. There are six strong Raman features, at 1558, 1531, 1298, 1161, 1064, and 785 cm^−1^, seen in the ns-TR^3^ spectrum of DN in the aqueous solution. The features at 1558 and 1531 cm^−1^ can mostly be attributed to C=N and C=C stretching vibrational modes. The 1298 cm^−1^ Raman feature can be attributed to the O–H rocking vibrational modes. The bands at 1161 cm^−1^, 1064 cm^−1^, and 785 cm^−1^ can be connected with the C–H wagging modes.

## 3. Materials and Methods

### 3.1. Materials

The sample of DN was synthesized following the literature procedure [10]. Spectroscopic grade MeCN were utilized for preparation of the sample solutions. All of the mixed solvent ratios are of volume ratios unless indicated otherwise.

### 3.2. Experimental and Computational Methods

#### 3.2.1. fs-TA and ns-TA Experiments

The fs-TA and ns-TA experiments were performed by following the same experimental apparatuses and methods that have been detailed previously [19,20,21]. In detail, fs-TA measurements were done using a femtosecond regenerative amplified Ti:sapphire laser system (Sarasota, FL, USA), in which the amplifier was seeded with the 120 fs laser pulses from an oscillator laser system. The laser probe pulse was produced by utilizing ~5% of the amplified 800 nm laser pulses to generate a white-light continuum (350–800 nm) in a moving CaF_2_ crystal, then this probe beam was split into two parts before going into the sample. One probe laser beam goes through the sample, while the other probe laser beam goes to the reference spectrometer in order to monitor the fluctuations in the probe beam intensity. For the present experiments, the sample solution was excited by a 267 nm pump beam (the third harmonic of the fundamental 800 nm from the regenerative amplifier). The 40 mL solutions were studied in a flowing 2 mm path-length cuvette with an absorbance of 1 at 267 nm throughout the data acquisition.

The ns-TA measurements were conducted using the commercial LP920 laser flash system (Livingston, UK). The fresh sample solutions were excited by a Q-switched Nd:YAG laser (4th harmonic line at λ = 266 nm). The probe light from a pulsed 450 W Xenon arc lamp was passed through various optical elements, samples, and a monochromator before being detected by a fast photomultiplier tube and recorded with a TDS 3012C digital signal analyzer). In the kinetics mode, a photomultiplier detector or InGaAs PIN detector is used, and the transient signal is acquired using a fast, high resolution oscilloscope. In the spectral mode, an array detector is fitted to the spectrograph exit port to measure a full range of wavelengths simultaneously. Unless specified otherwise, the ns-TA experiments were performed in air saturated solutions and the sample solutions were made up to have an absorbance of 1 at 266 nm.

#### 3.2.2. ns-TR^3^ Experiments

The ns-TR^3^ experiments have also been previously discussed in detail [21,22]. The pump laser pulse with a wavelength of 266 nm generated from the fourth harmonic of a Nd:YAG nanosecond pulsed laser, a 416 nm probe laser pulse produced from the first Stokes hydrogen Raman shifted laser line from the third (355 nm) harmonic, and a 341.5 nm probe laser pulse produced from the second Stokes hydrogen Raman shifted laser line from the fourth (266 nm) harmonic were employed in the ns-TR^3^ experiments. The two Nd:YAG lasers were synchronized electronically by a pulse delay generator to control the time delay of pump and probe lasers, and the time delay between the laser pulses was monitored by a fast photodiode and 500 MHz oscilloscope. The time resolution for this ns-TR^3^ experiments was approximately 10 ns. The pump and probe laser beams were lightly focused onto the sampling system, and the Raman light was collected using reflective optics into a spectrometer whose grating dispersed the light onto a liquid nitrogen cooled CCD detector. The spectra in this work were calibrated by the known MeCN solvent’s Raman bands with an estimated accuracy of (5 cm^−1^). The used samples solutions were prepared to have an UV absorption ~1 at 266 nm in a 1 mm path-length cuvette.

#### 3.2.3. Density Functional Theory Calculations

The DFT calculations were done employing the B3LYP hybrid functional with a 6-311G(d,p) basis set in PCM solvent mode with selected solvent molecules. No imaginary frequency modes were observed at the stationary states of the optimized structures. Time-dependent density functional theory was used to calculate the excitation energies, oscillator strengths, and the simulation of UV-vis spectra of selected intermediates. A Lorentzian function with a 10 cm^−1^ band width for the vibrational frequencies and a scaling factor of 0.975 were employed for comparison with experimental results [23,24]. More details could refer to our previous works [19,20]. The Gaussian 09 program suite [25] installed in HKU Research Computing facilities was employed.

## 4. Conclusions

The photochemistry of DN in MeCN and near neutral aqueous solutions was investigated by using fs-TA, ns-TA, and ns-TR^3^ spectroscopic methods, and results from DFT calculations were utilized to interpret the data. The formation of **2** was directly observed by fs-TA to take place within about 4 ps in MeCN and near neutral aqueous solution. Ns-TR^3^ experiments obtained information about the vibrational characteristic of **2** that exhibited a more intense resonance Raman signal than that for seen for **1** because of a halogen electronic effect. Ns-TA and ns-TR^3^ experiments examined the nucleophilic adduct reaction of **2** with H_2_O in a near neutral aqueous solution, and directly probed the formation of intermediate **3** that demonstrated the efficient and diagnostic reaction pathway for **2** with H_2_O when the DN para position is obstructed by the bromine atom. Scheme 2 shows the proposed reaction mechanisms for the photochemistry of DN in MeCN and in a near neutral aqueous solution as a summary. We expect that the results here could be useful in helping us understand and design the next time-resolved spectroscopic investigation of the covalent addition and/or other reactions between selected related nitrenium ions and other molecules.

## Supplementary Materials

The following are available online, including Figures S1–S5, as well as Cartesian coordinates, total energies, and vibrational zero-point energies from DFT calculations.
