# Supplementary material for: Time-Resolved Spectroscopic Study of N,N-Di(4-bromo)nitrenium Ions in Selected Solutions
Source: Molecules. 2018 Dec 3;23(12):3182. doi: 10.3390/molecules23123182 (PMC6321521; doi:10.3390/molecules23123182)
Supplement: Supplementary file 1 [file molecules-23-03182-s001.pdf]

Supporting Information

Article

Time-Resolved Spectroscopic Study of *N,N*-Di(4-bromo)nitrenium Ions in Selected Solutions

Lili Du [1,2,†], Xin Lan [1,†], Zhiping Yan [1], Ruixue Zhu[1,3] and David Lee Phillips [1,*]

1 Department of Chemistry, The University of Hong Kong, Hong Kong S.A.R., China; justailleen@gmail.com (L.D.); xinlan@connect.hku.hk (X.L.); mcayzp@gmail.com (Z.Y.); zhurx@shanghaitech.edu.cn (R.Z.)
2 Institute of Life Sciences, Jiangsu University, Zhenjiang 212013, China
3 School of Physical Science and Technology, Shanghai Tech University, Shanghai 201210, China
* Correspondence: phillips@hku.hk; Tel.: +852-2859-2160
† These authors contributed equally to this work.

Table of Contents

Figure S1. Shown are ns-TA spectra in obtained in pure MeCN solution after 266 nm irradiation of DN.1

Figure S2. The ns-TR^3 spectra produced in MeCN acquired by pump 266 nm, probe 416 nm. The star symbols indicate the regions affected by solvent subtraction artifacts. 2

Figure S3. The comparison between the DFT calculated normal Raman spectrum of nitrenium ions 2 and 1.. 3

Figure S 4. Fs-TA in 1:1 MeCN:H_2O solution acquired after 267 nm irradiation of DN (Left). Kinetics of the characteristic fs-TA absorption bands observed at 450 nm is shown in right. The solid lines indicate the kinetics fitting to the experimental data points. 3

Figure S5. Comparison of the ns-TR^3 10 ns spectrum achieved in MeCN (red) to one achieved in 1:1 MeCN: H_2O solution (black) by employing 416 nm as the probe and 266 nm as the pump of DN. The symbol * means features affected by solvent subtraction artifacts……………………………………………….…4

Cartesian coordinates, total energies, and vibrational zero-point energies…………………………………4

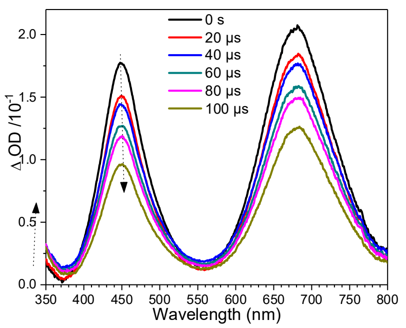

Figure S1. Shown are ns-TA spectra in obtained in pure MeCN solution after 266 nm irradiation for DN.

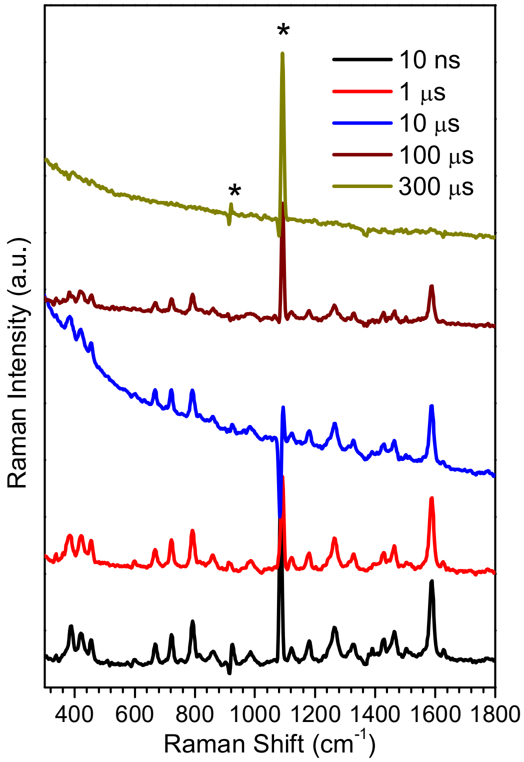

Figure S2. The ns-TR³ spectra produced in MeCN acquired by 266-nm pump and 416-nm probe of **DN**. The star symbols indicate the regions affected by solvent subtraction artifacts.

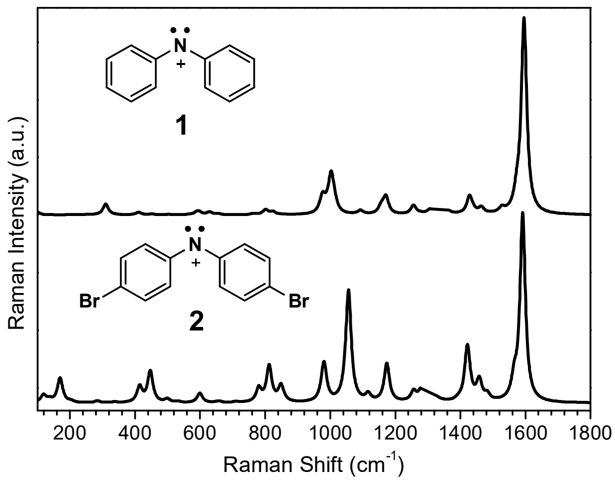

Figure S3. The comparison between the DFT calculated normal Raman spectrum of nitrenium ions **2** and **1**.

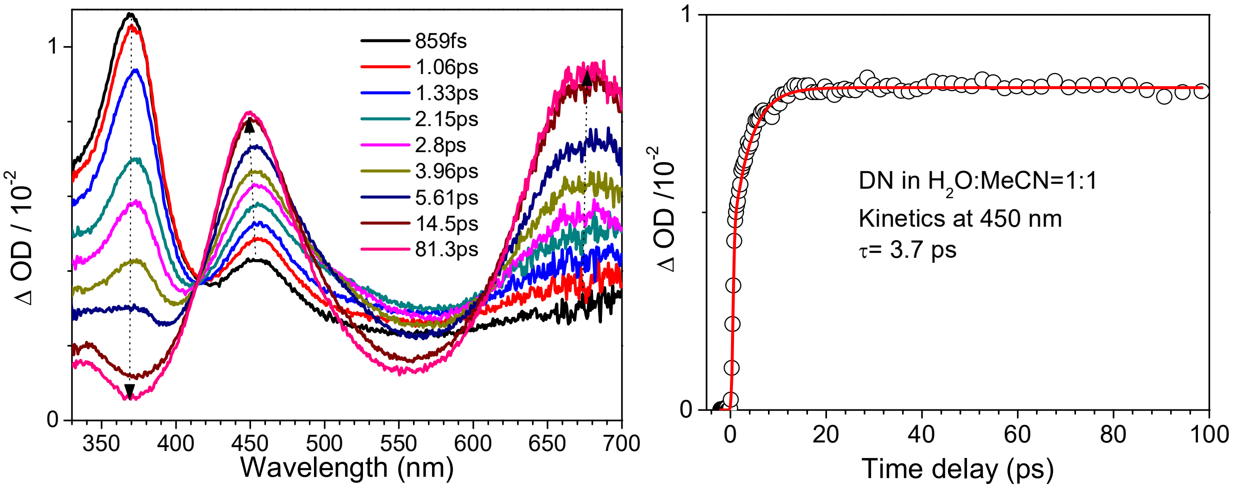

Figure S4. fs-TA in 1:1 MeCN:H_2O solution acquired after 267 nm irradiation of **DN** (left). Kinetics of the characteristic fs-TA absorption bands observed at 450 nm is shown in right. The solid lines indicate the kinetics fitting to the experimental data points.

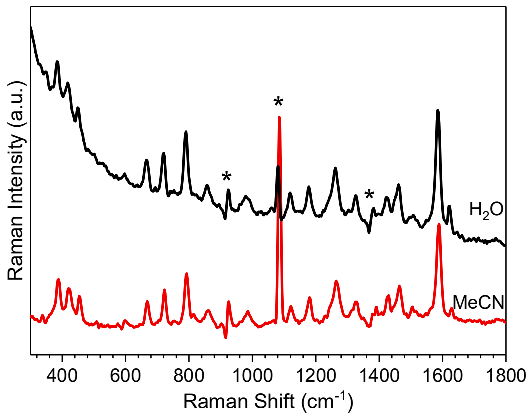

Figure S5. Comparison of the ns-TR[3] 10 ns spectrum achieved in MeCN (red) to one achieved in 1:1 MeCN: H_2O solution (black) by employing 416 nm as the probe and 266 nm as the pump of **DN**. The symbol * means features affected by solvent subtraction artifacts.

Cartesian coordinates, total energies, and vibrational zero-point energies for the optimized geometry from the (U)B3LYP/6-311G** calculations for the compounds and intermediates considered in this paper are given

Nitrenium ion **1**

C	-1.44443	-0.31987	-0.49905
C	-1.19313	1.01479	-0.04253
C	-2.30615	1.82637	0.34424
C	-3.57649	1.3048	0.39351
C	-3.77894	-0.02109	-0.02341
C	-2.72251	-0.823	-0.49387
N	0.	1.62176	0.
C	1.19314	1.01479	0.04253
C	1.44443	-0.31987	0.49906
C	2.72252	-0.823	0.49387
C	3.77894	-0.02109	0.02341
C	3.57649	1.3048	-0.39352
C	2.30615	1.82637	-0.34425
H	2.92204	-1.81761	0.86807
H	4.41071	1.90638	-0.72665
H	2.10729	2.84748	-0.64218
H	-2.10729	2.84748	0.64218
H	-4.41071	1.90638	0.72664
H	-2.92203	-1.81761	-0.86806
H	-0.64363	-0.90678	-0.92683
H	0.64364	-0.90677	0.92685

H	4.76988	-0.42465	0.01424
H	-4.76988	-0.42465	-0.01424

Zero-point correction= 0.185943 (Hartree/Particle)
Sum of electronic and thermal Free Energies= -517.796629 Hartree

Nitrenium ion **2**

C	1.46685	-0.35164	0.48677
C	1.19715	0.98359	0.03888
C	2.30407	1.80941	-0.33877
C	3.58121	1.30465	-0.3873
C	3.80578	-0.02203	0.02187
C	2.75096	-0.83693	0.48562
N	0.00004	1.5824	0.00014
C	-1.19736	0.98421	-0.0391
C	-1.46702	-0.35069	-0.48763
C	-2.75091	-0.83658	-0.4861
C	-3.80576	-0.022	-0.02192
C	-3.5813	1.30471	0.38738
C	-2.30429	1.80976	0.33885
Br	-5.54858	-0.70973	-0.00615
Br	5.54869	-0.70955	0.0064
H	-2.96572	-1.82943	-0.85878
H	-4.40993	1.91922	0.7132
H	-2.09114	2.83137	0.62718
H	2.09081	2.83112	-0.62663
H	4.40975	1.9194	-0.7129
H	2.96604	-1.82973	0.85829
H	0.67109	-0.95202	0.90707
H	-0.67138	-0.95093	-0.90865

Zero-point correction= 0.165275 (Hartree/Particle)
Sum of electronic and thermal Free Energies= -5664.906584 Hartree

Intermediate **3**

C	-2.32104	1.77636	0.49452
C	-1.07643	0.90695	0.32507
C	-1.26627	-0.53456	0.4543
C	-2.50176	-1.06821	0.34027
C	-3.67387	-0.23559	0.10218
C	-3.60315	1.09846	0.09469
N	0.00711	1.54376	0.07573
C	1.28009	0.94929	0.04561
C	1.82719	0.30064	1.16141
C	3.122	-0.20905	1.12297
C	3.87349	-0.07744	-0.03945

C	3.3542	0.57373	-1.15536
C	2.06804	1.09749	-1.1034
Br	5.65432	-0.78573	-0.10057
Br	-5.32024	-1.15127	-0.26435
H	-2.63538	-2.1428	0.36398
H	-4.4526	1.73608	-0.11229
H	-2.3996	1.90887	1.59614
H	1.65743	1.62251	-1.95744
H	3.95027	0.67995	-2.05245
H	3.53971	-0.70083	1.99196
H	1.24596	0.21953	2.07269
H	-0.39974	-1.17904	0.52203
O	-2.18895	3.02876	-0.12429
H	-1.23188	3.14609	-0.24509

Zero-point correction= 0.179585 (Hartree/Particle)
Sum of electronic and thermal Free Energies= -5740.933188 Hartree

Product **4**

C	1.46685	-0.35164	0.48677
C	1.19715	0.98359	0.03888
C	3.58121	1.30465	-0.3873
C	3.80578	-0.02203	0.02187
C	2.75096	-0.83693	0.48562
C	-1.19736	0.98421	-0.0391
C	-1.46702	-0.35069	-0.48763
C	-2.75091	-0.83658	-0.4861
C	-3.80576	-0.022	-0.02192
C	-3.5813	1.30471	0.38738
C	-2.30429	1.80976	0.33885
Br	-5.54858	-0.70973	-0.00615
Br	5.54869	-0.70955	0.0064
H	-2.96572	-1.82943	-0.85878
H	-4.40993	1.91922	0.7132
H	-2.09114	2.83137	0.62718
H	4.40975	1.9194	-0.7129
H	2.96604	-1.82973	0.85829
H	0.67109	-0.95202	0.90707
H	-0.67138	-0.95093	-0.90865
N	0.00004	1.5824	0.00014
H	-0.22203	2.16013	-0.7853
C	2.30407	1.80941	-0.33877
O	2.22316	3.06983	-0.65208
H	2.91291	3.55815	-0.19667

Zero-point correction= 0.181103 (Hartree/Particle)

Sum of electronic and thermal Free Energies= -5740.973898 Hartree
